# The effect of object processing in content-dependent source memory

**DOI:** 10.1186/1471-2202-14-71

**Published:** 2013-07-13

**Authors:** Heekyeong Park, Fernando Leal, Catherine Spann, Cheryl Abellanoza

**Affiliations:** 1 Department of Psychology, College of Science, University of Texas at Arlington, 501 S. Nedderman Drive, Arlington, TX 76019, USA

**Keywords:** Source memory, Content, Study processing, fMRI, Unitization

## Abstract

**Background:**

Previous studies have suggested that the study condition of an item influences how the item is encoded. However, it is still unclear whether subsequent source memory effects are dependent upon stimulus content when the item and context are unitized. The present fMRI study investigated the effect of encoding activity sensitive to stimulus content in source memory via unitization. In the scanner, participants were instructed to integrate a study item, an object in either a word or a picture form, with perceptual context into a single image.

**Results:**

Subsequent source memory effects independent of stimulus content were identified in the left lateral frontal and parietal regions, bilateral fusiform areas, and the left perirhinal cortex extending to the anterior hippocampus. Content-dependent subsequent source memory effects were found only with words in the left medial frontal lobe, the ventral visual stream, and bilateral parahippocampal regions. Further, neural activity for source memory with words extensively overlapped with the region where pictures were preferentially processed than words, including the left mid-occipital cortex and the right parahippocampal cortex.

**Conclusions:**

These results indicate that words that were accurately remembered with correct contextual information were processed more like pictures mediated by integrated imagery operation, compared to words that were recognized with incorrect context. In contrast, such processing did not discriminate subsequent source memory with pictures. Taken together, these findings suggest that unitization supports source memory for both words and pictures and that the requirement of the study task interacts with the nature of stimulus content in unitized source encoding.

## Background

Neural correlates of successful memory formation have been investigated with the functional magnetic resonance imaging (fMRI) subsequent memory procedure, in which neural activity during encoding for later remembered items is compared with activity for later forgotten items. Subsequent memory effects are often expressed as enhanced encoding activity for remembered items in several brain regions, such as the left prefrontal cortex, the fusiform area, and the medial temporal lobe (MTL) [1-3]. Further, subsequent memory tends to reveal the influence of the study condition in which the item is experienced at the cortical level. Seminal behavioral studies of memory have suggested that memory representation results from how an item is processed at encoding [4-6]. From this perspective, episodic memory is the by-product of study processing based on the circumstances in which an event is experienced. In accordance with this theoretical principle, previous fMRI studies of subsequent memory effects have shown that cortical encoding activity for successful memory of an item depends upon the nature of the study task, the modality of the study item, and the material of the study item [2,7-9].

In these studies, the differences in study conditions, including study task (semantic vs. non-semantic) or stimulus content such as modality (visual vs. auditory) and material (verbal vs. pictorial), dissociated the cortical regions where neural activity for successful memory formation was identified. For example, subsequent memory of words was predicted in the left inferior prefrontal cortex and the left fusiform region, whereas encoding activity for pictures was identified in the right inferior prefrontal cortex and bilateral fusiform areas [2]. The MTL effects of memory formation also showed left lateralized activation for words and right lateralized activation for pictures and faces [9]. Semantic encoding of successfully remembered words was related with activity in the left and medial prefrontal regions; however, non-semantic subsequent memory effects were found in the right prefrontal cortex, bilateral intra-parietal sulci and bilateral fusiform gyri [7]. Moreover, subsequent memory effects for visually presented words were reported in the right fusiform cortex, while auditory subsequent memory effects were identified in bilateral superior temporal sulci [10].

However, relatively little is known about whether the formation of associations, as opposed to encoding of single items, also exhibits study condition-specific effects of encoding. The extant findings of task-dependent effects in memory associations are less consistent. For example, a study that compared task effects on associative memory of word pairs using semantic and phonological tasks found that extensive activity in the left inferior prefrontal cortex predicted word pairs that were later successfully remembered as an intact pair in both encoding tasks; task-specific effects, however, were negligible [11]. Another study that compared semantic versus perceptual study conditions for associative memory of word pairs reported that the same left inferior prefrontal regions were involved in subsequent associative memory effects common to both conditions; however, perceptual associative encoding effects were also found in the left temporo-occipital and bilateral parietal cortices as well as the right parahippocampal cortex [12].

Other studies of subsequent source memory (item-source associations) showed that neural correlates of source memory differed depending upon study task or stimulus content [13,14]. Subsequent source memory of words studied with a semantic task was localized in the left temporo-occipital cortex, while successful source memory effects with a non-semantic encoding task were found in the right temporo-occipital region, revealing hemispheric differences between subsequent source memory effects that depend upon the study task [13]. Subsequent source memory effects also varied by stimulus content, such that source encoding activity for words were found in the left inferior prefrontal cortex, while successful source memory effects for objects were identified in the left perirhinal cortex [14].

In addition to these cortical effects, the MTL is known to be critical for forming associations [12,15,16]. From the memory process view, the MTL subregions are specialized for different memory processes. That is, the hippocampus and the parahippocampal cortex are integral to the formation of associations based on recollection, whereas the perirhinal cortex tends to support familiarity-based item recognition [17-21]. Functional dissociation within the MTL for different memory processes has been supported by findings that increased activity in the hippocampus predicts subsequent recognition based on recollection (e.g., source memory) but not familiarity [22-24], whereas familiarity-based judgments (e.g., item memory) have been related with increased perirhinal activity during encoding [18,22,23]. It was further proposed that the perirhinal cortex could support source memory based on familiarity through unitization of an item and context [25]. As opposed to the conventional way of binding (i.e., non-unitized encoding), in which separate item and context are to be combined through associations, unitization is assumed to bind context to the item as an item feature. From this view, source memory through unitization may be supported by familiarity. Further, source memory via unitization was supported by perirhinal activity, which is consistent with the proposal of the perirhinal cortex for familiarity-based source recognition [25,26].

It has also been suggested that MTL subregions are involved in encoding of heterogeneous classes of stimulus content [22,27]. First, the hippocampus is involved in processing of content-independent binding. For instance, encoding activity in the anterior hippocampus was identified for recollection of both objects and scenes, reflecting the content-independent role of the hippocampus [28]. Other MTL subregions selectively engage in processing of different types of content. Specifically, the parahippocampal cortex is selective to visuo-spatial processing such as scene processing [29-33]. The perirhinal cortex is involved in object processing, and the role of perirhinal cortex in object encoding was found across different recognition judgments (i.e., Remember, Know, and New) and source memory [29]. Similarly, a MTL subsequent memory study that examined source encoding with objects and scenes reported the dissociation of the MTL cortex by content: the perirhinal cortex contributed to source encoding for objects, but the parahippocampal cortex supported source encoding of scenes [32].

Though previous studies suggested content-dependent encoding effects, most of those studies examined content-dependent effects with non-unitized encoding tasks or focused on MTL activity. As alluded to earlier, unitization of an item and context may change the way that the item and context are bound; therefore, unitized source memory may be supported by different encoding activity at the cortical level. Considering that the memory process involved in unitized encoding may be different from the memory process for non-unitized encoding, an interesting question is whether subsequent memory effects specific to stimulus content would appear with unitized encoding both at the cortical and MTL levels. This is important, as it investigates how encoding activity specific to each trial (i.e., stimulus content) is influenced by study processing across trials (i.e., encoding operation) in forming associations. Previous studies that used non-unitized encoding tasks reported content-independent effects in the hippocampus and content-dependent effects in the cortical areas. However, it is not clear whether unitized encoding is sensitive to content-dependent subsequent memory effect. Additionally, given that non-unitized encoding tends to elicit hippocampal activity for source memory formation across different types of content, it is of interest whether unitized encoding would show similar MTL source memory effects across verbal and pictorial content. In what follows, we report a study of the formation of source memory for words and pictures via unitization.

Participants were presented a list of study items (i.e. words and pictures) on a colored background and were instructed to imagine the item in the background color. This meant that participants actively had to generate a mental presentation for words but not necessarily for pictures. Thus, imagery-based operations required by the study task would have different effects depending on whether the content was verbal or pictorial. By comparing encoding activity of words and pictures varied by later source memory performance (source correct vs. source incorrect), we investigated the effect of imagery-based operations in source memory by stimulus content.

Previous studies of subsequent memory effects have reported encoding activity for perceptual processing of visually-presented stimuli in the parietal and occipital cortices, as well as the fusiform cortex [10,13,34-36]. Further, perirhinal activity has been reported for the formation of source memory with objects through unitization [29,31,32]. Thus, it is likely that parietal/occipital activity as well as perirhinal activity would contribute to source encoding of both types of content. On the other hand, participants had to generate a visuo-spatial representation of the word and place it in the color context for words, whereas pictures were already presented in the necessary visual form. That said, visuo-spatial processing and binding would be critical for forming successful source memory with words by engaging in the study task, but less so with pictures. Then, neural activity for visuo-spatial processing and binding would distinguish words with contextual information from words without correct context to a greater extent than such activity with pictures, which would emerge as content-dependent subsequent source memory effects. Further, as pictures were presented in the pictorial form during study, the brain regions that showed picture-preferential processing (pictures > words) regardless of later source memory effects would be the areas where subsequent source memory effects selective to words would be identified.

As such, we hypothesized the following for the present experiment:

(1) For subsequent source memory effects independent of stimulus content, unitization of item and color would be supported by encoding of visually presented objects, as evidenced by increased activity in the parieto-occipital cortices, the fusiform gyrus, and the perirhinal cortex, in addition to the established role of hippocampal activity in source memory formation overall.

(2) Study requirement for engaging in visuo-spatial processing and binding for source memory formation would affect words more than pictures, and this would call for greater subsequent source memory effects selective to words in the ventral visual pathway and the parahippocampal cortex.

(3) Subsequent source memory effects for words would indicate that these words were processed more like pictures, based on the overlap of regions where pictures were preferentially processed compared to words such as parietal and occipital regions.

## Results and discussion

### Behavioral results

Proportions of recognition judgments (source correct, source incorrect, item miss) by stimulus content (word, picture) are displayed in Table 1. The proportions of correct source judgments for both stimulus contents were significantly greater than the chance level (.25), *t*s _[_23_]_ > 15.82, *ps* < .001. There was no main effect of stimulus content, but there was a significant interaction between content and recognition judgment, *F*_[_2_,_46_]_ = 14.42, *p* < .001. Follow-up tests showed that more source correct judgments were made with words than pictures, *t*_[_23_]_ = 2.34, *p* < .05, whereas more source incorrect judgments were made with pictures than words, *t*_[_23_]_ = 5.26, *p* < .001, although item misses were greater with words than pictures, *t*_[_23_]_ =3.09, *p* < .005. Further, using *d’* as a measure of recognition accuracy, more accurate source judgments were made for words (1.73 [.15]) than for pictures (1.27 [.17]), *t*_[_23_]_ = 4.28, *p* < .001.

**Table 1:** Mean test response proportions (SEM) and mean study reaction times (in ms) as a function of stimulus content and memory judgment

**Stimulus content**	**Studied**			**New**	
	**Source correct**	**Source incorrect**	**Miss**	**Correct rejection**	**False alarm**
Word	.69 (.03)	.19 (.02)	.12(.02)	.91 (.03)	.09 (.03)
	1656 (84)	1671 (80)	1661 (83)		
Picture	.66 (.02)	.26 (.02)	.08 (.01)	.93 (.01)	.07 (.01)
	1675 (84)	1733 (83)	1767 (74)		

Study reaction times are also shown in Table 1, segregated by stimulus content and later source memory judgment. A 2 × 2 ANOVA with stimulus content (word, picture) and source judgment (source correct, source incorrect, item miss) as factors was conducted on these data. The ANOVA showed the main effect of stimulus content, indicating longer reaction times for pictures than for words, *F*_[_1_,_23_]_ = 8.59, *p* < .005. However, there was neither a main effect of source judgment nor an interaction between stimulus content and source judgment on study times.

### fMRI Results

Analyses of subsequent source memory effects were based on contrasts between encoding activity of study items that were later accurately endorsed with the studied color (source correct) as opposed to encoding activity of study items that were later recognized but with an incorrect color (source incorrect). Item misses were not included in the analysis due to an insufficient number of trials.

### Neural activity for source memory formation independent of stimulus content

**Figure 1:**
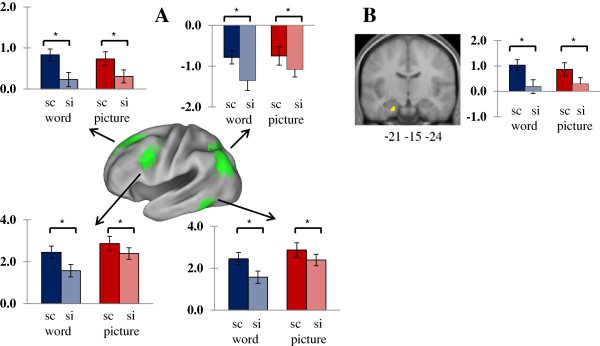
**Content-independent subsequent source memory effects. A:** Lateral content-independent subsequent source memory effects. **B:** MTL content-independent effects overlaid on mean anatomical image across subjects. Mean parameter estimates elicited by stimulus content (sc: source correct, si: source incorrect, *p*s < .05).

First, we sought neural activity associated with the formation of source memory independent of stimulus content via unitization. In order to find subsequent source memory effects, the statistical parametric mappings (SPMs) for the main effect of source memory across stimulus content (source correct > source incorrect) were exclusively masked with the SPMs for the interaction of source memory by content. The outcome of this analysis is shown in Table 2. Source encoding activity was localized exclusively in the left hemisphere, including superior to inferior lateral parts of the prefrontal cortex, the orbito-frontal region, the lateral parietal and occipital cortices, and the fusiform gyrus (Figure 1A). Source memory independent of stimulus content was also identified in the left perirhinal cortex extending to the left hippocampus (Figure 1B). The mean parameter estimates extracted from all of these clusters demonstrated that encoding activity was greater for source correct than for source incorrect items, and these differences were independently reliable for both stimulus content from all clusters (*p*s < .05). The contrast in the opposite direction (source incorrect > source correct exclusively masked with the interaction) did not reveal any suprathreshold clusters.

**Table 2:** Subsequent source memory effects independent of stimulus content

**Coordinates (x y z)**			***Z *(# of voxels)**		**Region**	**BA**
Non-MTL effects						
−12	48	42	4.42	104	L superior frontal gyrus	8/9
−42	9	36	4.30	56	L inferior frontal gyrus	44
−9	9	−21	4.25	39	L inferior orbito-frontal cortex	47
−42	−81	33	4.47	156	L angular gyrus/Inferior/superior parietal lobule/ Mid occipital cortex	19/39
−54	−57	−21	3.52	39	L fusiform gyrus	37
MTL effects						
−21	−15	−24	3.84	39	L perirhinal cortex/anterior hippocampus	

### Neural activity for source memory formation depending on stimulus content

**Figure 2:**
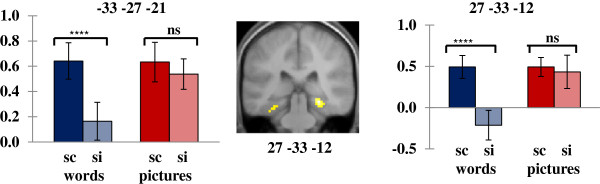
**Word-dependent subsequent source memory effects in the MTL.** Effects are overlaid on a section (y = −33) of the across-subject mean anatomical image.

Next, we looked for encoding activity for source memory varied by the content of the stimulus through integrated encoding of item and context. Subsequent source memory effects dependent on content were identified by exclusively masking source memory effects in one stimulus content type (e.g., source correct > source incorrect with word trials and vice versa) with subsequent source memory effects in the alternate content type (e.g., source correct > source incorrect with picture trials and vice versa). This procedure revealed only subsequent source memory effects selective to words, which occurred in the superior medial parts of the left frontal lobe, the right insula, and the left ventral visual stream (Table 3). As illustrated in Figure 2, MTL source memory effects selective to words were identified in bilateral middle/posterior parahippocampal cortices, and activity in the right parahippocampal cortex extended to the vicinity of the right hippocampus. ANOVAs on parameter estimates extracted from all clusters showing word-dependent source memory effects revealed significant interactions between stimulus content and source memory judgment, *F*s_[_1_,_23_]_ > 5.8, *p*s < .05. Follow-up tests revealed that words that were subsequently remembered with correct source information showed greater encoding activity in these regions than words recognized with incorrect source information (*p*s < .001), whereas pictures did not differ by source memory in these regions. Subsequent source memory effects selective to pictures did not reveal a single cluster over the threshold.

**Table 3:** Subsequent source memory effects selective to words

**Coordinates (x y z)**			***Z *(# of voxels)**		**Region**	**BA**
Non-MTL effects						
−15	36	30	3.59	27	L medial frontal gyrus	32
−24	27	54	4.25	57	L middle frontal gyrus	8
27	3	24	4.06	40	R insula/caudate	
−3	−3	15	3.64	41	Thalamus	
−42	−57	−18	3.86	49	L fusiform gyrus	37
−42	−69	9	4.27	77	L middle occipital gyrus	39
MTL effects						
−33	−27	−21	3.61	17	L parahippocampal cortex	36
27	−33	−12	4.39	57	R parahippocampus/ hippocampus	

### Overlap between subsequent source memory effects and processing effects of stimulus content

**Figure 3:**
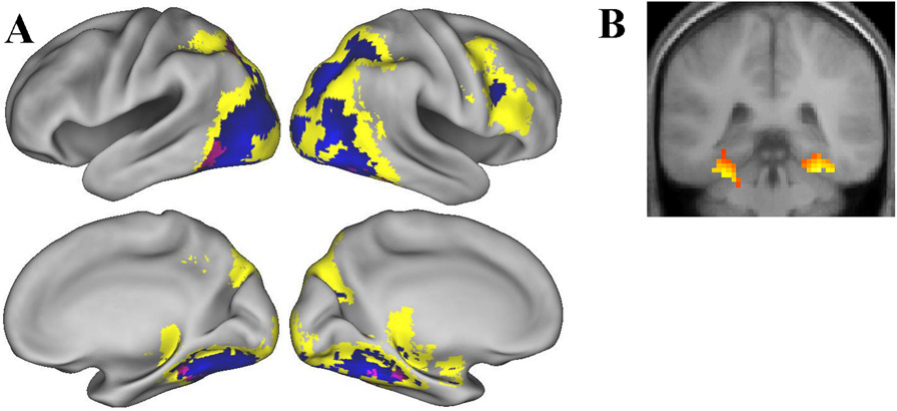
**Overlap between content-dependent subsequent source memory effects and content-preferential processing areas. A:** Cortical regions where subsequent source memory effects elicited by words (blue) and pictures (red) overlap with regions where activity is greater on picture trials than word trials (thresholded at *p* < .001, yellow). Areas of overlap between words and pictures are shown in purple. **B:** Overlap between word-dependent source memory effects and picture processing in bilateral parahippocampal cortices.

We also examined whether subsequent source memory effects depending on content overlapped with brain regions where corresponding stimulus content trials elicited more activity than alternate content trials. Subsequent source memory effects for one content type (e.g., source correct > source incorrect for words and vice versa) were inclusively masked with corresponding effects for content processing (e.g., words > pictures and vice versa). No overlap was found either with word or picture source memory effects in the area where words had elicited more activity than pictures. Rather, subsequent source memory effects for both words and pictures did overlap with brain regions where pictures had elicited more activity than words (Figure 3). Source encoding activity for pictures was overlapped with picture-preferential processing areas in the bilateral fusiform areas and the left superior parietal lobe. Extensive overlap was found between word source encoding activity and picture processing in bilateral parahippocampal cortices and occipital regions.

### Discussion

The present study investigated content-dependent source memory effects by means of an integrated imagery operation. Successful source memory that did not differ between the two stimulus content types was predicted in the left inferior frontal gyrus (LIFG), the lateral parietal/occipital lobes, and the fusiform gyrus. In addition, MTL subsequent memory effects independent of stimulus content were identified in the left perirhinal cortex extending to the left anterior hippocampus. The formation of source memory selective to words recruited activity in the left frontal cortex, the ventral visual stream, and the bilateral parahippocampal cortices extending to the vicinity of the right posterior hippocampus. Picture-dependent subsequent source memory effects did not emerge over threshold. Finally, subsequent source memory effects for words extensively overlapped with the areas where pictures were preferentially processed.

Content-independent subsequent source memory effects were identified in a number of cortical regions. First, subsequent source memory effects were evident in the LIFG extending to the superior frontal regions. LIFG effects have been found in the formation of associative memory [11,12,37,38] and source memory [14,34] with both semantic and non-semantic encoding tasks. The current finding of LIFG involvement in integrated source encoding is consistent with the proposal that the LIFG is crucial for the formation of associations regardless of the type of study processing [11,38]. Encoding activity for source memory independent of stimulus content was also found in the left lateral parietal cortex, spanning from the angular gyrus to the superior parietal/occipital regions. These regions, especially the intra-parietal sulcus, are known to engage in goal-directed attentional processing [39,40], multi-modal processing [35,41], and perceptual processing [35,36,42], in addition to encoding of both item and source memories [7,10,18,43]. Previous studies also reported subsequent source memory effects in the LIFG and the left parietal regions with non-unitized encoding tasks. Thus, the present findings of the LIFG and the left parietal activity through integrated binding of item and context indicates that neural activity in these regions supports the formation of successful source memory mediated by perceptual processing through unitized encoding as well as non-unitized encoding.

In parallel with these cortical effects, content-independent subsequent source memory effects were identified in the left perirhinal cortex extending to the left anterior hippocampus, which is consistent with previous reports of perirhinal activity for source memory formation with objects [14,29,33] and prior findings of hippocampal activity for source encoding in general [12,31,32]. The formation of associations via unitization of an item and its contextual details is known to be supported by perirhinal activity, as the unitized representation of the item with context as an object can be recognized based on familiarity [44,45]. The current finding of perirhinal activity in source encoding with both words and pictures extends previous findings of perirhinal involvement in associations [18,19,32,33,46] in that source memory for both verbal and pictorial content is supported by perirhinal activity through unitization. Further, activity in the hippocampus has been found in subsequent source memory in a content-general manner, which confirms the established role of the hippocampus in memory associations overall [19,28,31].

While investigating content-dependent subsequent source memory effects, we found only word-dependent effects. These effects were evident in the visual ventral stream, the region critical for object identification (i.e., the “what” pathway), which suggests that accurate source memory of words was mediated by visuo-spatial object processing more so than inaccurate source memory of words. That is, words that were subsequently remembered with source information tended to be the ones with more object-like processing during encoding, whereas words that were not accompanied with correct source information tended to be the ones that were not visualized well enough for imagery operation to occur at a level comparable to those that elicited source correct judgments.^a^ However, the level of object processing did not distinguish subsequent memory of pictures between source correct versus source incorrect. Considering that pictures were presented in the visual object form during encoding, object processing would likely occur for all pictures regardless of whether they were accompanied with correct contextual information or not, hence the smaller impact of object processing in subsequent source memory effects with pictures. This interpretation is supported by the significant interaction between stimulus content and source judgment revealing the differences in parameter estimates only with source correct words versus source incorrect words (see Results). In addition, insular activity findings complement previous findings of insular involvement in multi-modal processing of stimuli [42] as well as source encoding [34,47].

Word-dependent source memory effects were also identified in the bilateral parahippocampal cortices, extending to the vicinity of the right posterior hippocampus. These MTL effects reflect that activity of word-dependent source memory exhibits not only extensive object processing but also additional visuo-spatial and contextual processing for the words that were later remembered with correct source, compared with words without correct source, consistent with prior reports of parahippocampal activity for both spatial and nonspatial contexts [46,48]. On the other hand, there was no overlap between word-dependent source memory effects and the region where words were preferentially processed than pictures. Prior studies have shown overlap of content-dependent subsequent memory effects in the areas where corresponding content are preferentially processed [10,38,43]. The current, seemingly contradictory findings illustrate that successfully remembered words with accurate context are indeed the outcome of encoding in more of an ‘object’ manner due to the requirement of the study task. These findings suggest that adopting pictorial processing by engaging in the imagery-based encoding task was crucial for the formation of successful source memory only with words due to the interaction between the nature of study processing and the stimulus content.^b^ In sum, the present findings demonstrate that the requirement of trial-general study processing influences the way that trial-specific stimulus content is experienced. Importantly, the present findings show that unitization is supported for different types of stimulus content.

## Conclusions

We investigated neural activity specific to stimulus content via integrated source encoding with words and pictures. As predicted, subsequent source memory effects were evident in the left lateral frontal and parietal cortex as well as the left perirhinal cortex extending to the hippocampus, which is indicative of the significance of these regions in source memory formation for integrated object-context. Content-dependent source memory effects were found only with words in the ventral visual stream and the parahippocampal cortex, which reflects more visuo-spatial processing and contextual binding with words for successful source memory due to the requirement of imagery encoding operation. Collectively, the present findings demonstrate the effect of trial-general study processing in subsequent source memory effects depending on stimulus content through unitization.

## Methods

### Subjects

Twenty-four subjects participated in the experiment (16 females; 18–29 years). They were recruited from the University of Texas−Arlington community and compensated for their participation. All subjects reported being right−handed, being native English speakers, and having no history of neurological disease. Informed consent was obtained prior to participation. The experiment was approved by the University of Texas−Arlington and University of Texas Southwestern Medical Center Institutional Review Boards.

### Materials

The experimental stimulus pool consisted of 175 concrete words (4–9 letter nouns) and 175 line-drawings depicting nameable objects. Three-hundred critical items were selected from the stimulus pool to construct the study and test lists. For each subject, a study list of 200 critical items (100 words and 100 line-drawing pictures) was created from the pool by random selection of items without replacement. Critical items were pseudo-randomly assigned to one of four color backgrounds (blue, green, red, or yellow), 25 words, and 25 pictures for each color. All words were presented in black, all pictures were presented in black-white line-drawing form, and both words and pictures were overlaid on the colored background. Each study list comprised an intermixed presentation of critical items with 100 null trials interspersed among the experimental trials. A test list was consisted 200 studied items intermixed with 100 new items (50 words and 50 pictures). Test items were presented on a gray background. Both study and test list sequences were constrained by no more than four consecutive presentations of a type of content or background color. An additional 50 items, 25 words, and 25 pictures were used for practice trials. The composition and ordering of the study and test lists were made anew for each subject.

All items were projected onto a screen viewed by subjects via a mirror mounted on the scanner head. For the study list, critical items were presented on one of four color backgrounds, while null events were presented on a gray background. For the test list, all items were presented on the gray background. All items were presented at a maximum visual angle of 7.1° × 7.1°.

### Procedure

Subjects were given instructions and practice for experimental tasks prior to the experiment proper. The experiment consisted of two study-test cycles. For each study phase, a fixation cross was presented for 200 ms as a warning signal for the upcoming item, followed by a study item superimposed on the colored background for 2000 ms. The study item was replaced by a response prompt (‘+’) for 1300 ms prior to the onset of the next trial. Stimulus onset asynchrony was 3500 ms. Subjects were instructed to form an image of the object (e.g., ‘UMBRELLA’) in the background color (e.g., ‘RED’) and to make a pleasantness judgment for the colored object (‘RED UMBRELLA’). Note that the pleasantness judgment was made based on the mental image that integrated object and color. This study instruction was aimed to facilitate unitization of the object and color context. Subjects indicated their judgments by pressing a button with the index or middle finger of their right hand. The finger assignment to each response was counterbalanced across subjects. Four colors (blue, green, red, or yellow) were used for backgrounds, and 50 items (half words and half pictures) were presented in each colored background. For null trials, a fixation character (‘+’) was continuously displayed for 3500 ms on a gray background, and no response was required.

The test phase followed approximately 5 min after the end of the study phase. Test items were presented on the gray background. The content of the stimulus was kept consistent between study and test (e.g., if a stimulus was studied in a word form, it was also tested in the word form). Subjects were asked to judge whether the test item was previously studied in the experiment and, if it had been studied, to indicate in which color it was presented; subjects responded using a one-step source recognition response. Each study and test session lasted about 20 minutes.

### fMRI scanning

A Philips Achieva 3T MR scanner (Philips Medical Systems, Andover, MA) fitted with a 32-channel RF receiver head coil was used to acquire both T_1_-weighted anatomical volume images (256 × 238 matrix, 1 mm^3^ voxels) and T^*^_2_-weighted echo-planar images (EPIs) (80 × 80 matrix, 3 mm × 3 mm in-plane resolution, axial acquisition, flip angle 70°, TE 30 ms) per volume. Each EPI volume comprised thirty 3 mm-thick axial slices acquired in a descending sequential order and separated by 1 mm, providing coverage of almost the entire brain. Data were acquired during the study and test phases in four scanning sessions comprising 270 volumes each, with a repetition time (TR) of 2 s. Five additional volumes were collected at the beginning of each run, but these were discarded to allow for T_1_ equilibration. The 3.5 s SOA allowed for an effective sampling rate of the hemodynamic response of 2 Hz.

### fMRI data analysis

Data preprocessing and statistical analyses were performed with Statistical Parametric Mapping (SPM 8, Wellcome Department of Cognitive Neurology, London, UK: http://www.fil.ion.ucl.ac.uk), implemented in MATLAB 9 (Mathworks, Natick, MA). For each subject, functional images were registered to the first image of each scan session and temporally realigned to the middle slice using sinc interpolation. The resulting data were normalized to a standard EPI template based on the Montreal Neurological Institute (MNI) reference brain and resampled into 3 mm isotropic voxels using nonlinear basis functions [49]. The normalized images were smoothed with an isotropic 8 mm full-width half-maximum Gaussian kernel. The time series in each voxel were high-pass filtered to 1/128 Hz to remove low-frequency noise and scaled to a grand mean of 100 across both voxels and scans. T_1_-weighted anatomical images were coregistered to the mean EPI volume and normalized to a standard T_1_ template of the MNI brain. After normalization, an across-subject mean anatomical image was created as the study-specific template for identifying brain regions.

Statistical analysis was performed on the study phase data using a two-stage mixed effects model for investigating encoding activity associated with content-dependent source memory effects through unitization of the item and context. In the first stage, neural activity was modeled by delta functions at stimulus onset. The event-related blood oxygen-level dependent (BOLD) response was modeled by convolving these neural functions with a canonical hemodynamic response function (HRF) and its temporal and dispersion derivatives. In addition, six regressors were modeled for movement-related variance, and session-specific constant terms were employed to model the mean image intensity in each session. Parameter estimates for events of interest were measured for each subject using a General Linear Model. Non-sphericity of the error covariance was accommodated by an AR_(1)_ model in which the temporal autocorrelation was estimated by pooling over suprathreshold voxels [50]. The parameters for each covariate and the hyperparameters governing the error covariance were estimated using Restricted Maximum Likelihood (ReML). In the second stage, linear contrasts of these subject-specific parameter estimates were computed, treating subjects as a random effect.

For the analysis of subsequent source memory effects by stimulus content, four events of interest were defined: ‘word-source correct’ (studied words that were recognized with correct study color); ‘word-source incorrect’ (studied words that were recognized albeit with incorrect study color); ‘picture-source correct’ (studied pictures that were accurately judged with study color); and ‘picture-source incorrect’ (studied pictures that were responded with incorrect color). All other study trials including item misses (studied items that were incorrectly judged as new) and no responses were modeled as events of no-interest, due to an insufficient number of trials.^c^

At the whole brain level, only effects surviving a voxel-wise threshold of *p* < .001 and a corrected cluster-wise threshold of *p* < .05 (greater than 16 voxels) were interpreted. When exclusive masking was applied to identify voxels where effects were not shared between two contrasts, the mask threshold was set at a two-tailed threshold of *p* < .1. Note that the more liberal the threshold of an exclusive mask, the more conservative the masking procedure. The threshold for the inclusive mask was set to *p* < .01. The peak voxels of clusters exhibiting reliable effects are reported in MNI coordinates. For the regions identified from main analyses, region-specific parameter estimates were extracted by peak voxel activity of a cluster for each subject and subjected to group-level statistical tests. The significance level for the region-specific parameter estimates was set to *p* < .05.

## Endnotes

^a^We thank Reviewer for clarification of this interpretation.

^b^At the MTL level, subsequent source memory effects dependent on words may be accounted by recollection. That is, words that were recognized with correct source information recruited more activity related with recollection process in the parahippocampal cortex and the hippocampus due to binding of the item and context, compared with words without source memory. To put it differently, source correct words were more likely the words that were accompanied with the recollection process of binding the item and context compared with source incorrect words, whereas subsequent source memory dependent on pictures was not significantly distinguished by recollection process. We thank Reviewer for this suggestion.

^c^Only 7 out of 24 participants had sufficient numbers of trials (> 10) for both words and pictures.

## Competing interest

The authors declare that they have no competing interests.

## Authors’ contributions

HP designed the study, participated in data collection, performed the statistical analysis, and wrote the manuscript. FL and CS participated in data collection. CA participated in writing the manuscript. All authors read and approved the manuscript.

## References

[B1] Wagner AD Schacter DL Rotte M Koutstaal W Maril A Dale AM Rosen BR Buckner RL Building memories: Remembering and forgetting of verbal experiences as predicted by brain activity Science 1998 281 1188 1191 9712582 10.1126/science.281.5380.1188

[B2] Kirchhoff BA Wagner AD Maril A Stern C Prefrontal-temporal circuitry for episodic encoding and subsequent memory J Neurosci 2000 20 6173 6180 10934267 10.1523/JNEUROSCI.20-16-06173.2000 PMC6772579

[B3] Paller KA Wagner AD Observing the transformation of experience into memory Trends Cogn Sci 2002 6 93 102 10.1016/S1364-6613(00)01845-3 15866193

[B4] Craik FIM Lockhart RS Levels of processing: A framework for memory research J Verb Learn Verb Be 1972 11 671 684 10.1016/S0022-5371(72)80001-X

[B5] Craik FIM Tulving E Depth of processing and the retention of words in episodic memory J Exp Psychol Gen 1975 104 268 294

[B6] Hyde TS Jenkins JJ Recall for words as a function of semantic, graphic, and syntactic orienting tasks J Verb Learn Verb Be 1972 12 471 480

[B7] Otten LJ Rugg MD Task-dependency of the neural correlates of episodic encoding as measured by fMRI Cereb Cortex 2001 11 1150 1160 10.1093/cercor/11.12.1150 11709486

[B8] Mitchell KJ Johnson MK Raye CL Greene EJ Prefrontal cortex activity associated with source monitoring in a working memory task J Cogn Neurosci 2004 16 921 934 10.1162/0898929041502724 15298780

[B9] Powell HW Koepp MJ Symms MR Boulby PA Salek-Haddadi A Thompson PJ Duncan JS Richardson MP Material-specific lateralisation of memory encoding in the medial temporal lobe: Blocked verus event-related design Neuroimage 2005 27 231 239 10.1016/j.neuroimage.2005.04.033 15927485

[B10] Gottlieb LJ Rugg MD Effects of modality on the neural correlates of encoding processes supporting recollection and familiarity Learn Memory 2011 18 565 573 10.1101/lm.2197211 PMC3256569 21852431

[B11] Park H Rugg MD Neural correlates of successful encoding of semantically and phonologically mediated inter-item associations Neuroimage 2008 43 165 172 10.1016/j.neuroimage.2008.06.044 18675362 PMC2575045

[B12] Prince SE Daselaar SM Cabeza R Neural correlates of relational memory: successful encoding and retrieval of semantic and perceptual associations J Neurosci 2005 25 1203 1210 10.1523/JNEUROSCI.2540-04.2005 15689557 PMC6725951

[B13] Park H Uncapher M Rugg MD Effects of study task on the neural correlates of encoding operations supporting successful source memory Learn Memory 2008 15 417 425 10.1101/lm.878908 PMC2414252 18511693

[B14] Duarte A Henson RN Graham KS Stimulus content and the neural correlates of source memory Brain Res 2011 1373 110 123 21145314 10.1016/j.brainres.2010.11.086 PMC3098368

[B15] Summerfield C Greene M Wager T Egner T Hirsch J Mangels J Neocortical connectivity during episodic memory formation PLoS Bio 2006 4 e128 10.1371/journal.pbio.0040128 16605307 PMC1436028

[B16] Qin S Piekema C Petersson KM Han B Luo J Fernandez G Probing the transformation of discontinuous associations into episodic memory: an event-related fMRI study Neuroimage 2007 38 212 222 10.1016/j.neuroimage.2007.07.020 17804259

[B17] Davachi L Mitchell JP Wagner AD Multiple routes to memory: Distinct medial temporal lobe processes build item and source memories P Natl Acad Sci USA 2003 100 2157 2162 10.1073/pnas.0337195100 PMC149975 12578977

[B18] Ranganath C Yonelinas AP Cohen MX Dy CJ Tom SM D’Esposito M Dissociable correlates of recollection and familiarity within the medial temporal lobes Neuropsychologia 2003 42 2 13 14615072 10.1016/j.neuropsychologia.2003.07.006

[B19] Greve A Evans CJ Graham KS Wilding EL Functional specialisation in the hippocampus and perirhinal cortex during the encoding of verbal associations Neuropsychologia 2011 49 2746 2754 10.1016/j.neuropsychologia.2011.06.002 21683723

[B20] Brown MW Aggleton JP Recognition memory: what are the roles of the perirhinal cortex and hippocampus? Nat Rev Neurosci 2001 2 51 61 10.1038/35049064 11253359

[B21] Eichenbaum H Yonelinas AR Ranganath C The medial temporal lobe and recognition memory Annu Rev Neurosci 2007 30 123 152 10.1146/annurev.neuro.30.051606.094328 17417939 PMC2064941

[B22] Davachi L Item, context and relational episodic encoding in humans Curr Opin Neurobiol 2006 16 693 700 10.1016/j.conb.2006.10.012 17097284

[B23] Kensinger EA Schacter DL Amygdala activity is associated with the successful encoding of item, but not source, information for positive and negative stimuli J Neurosci 2006 26 2564 2570 10.1523/JNEUROSCI.5241-05.2006 16510734 PMC6793660

[B24] Yonelinas AP Otten LJ Shaw KN Rugg MD Separating the brain regions involved in recollection and familiarity in recognition memory J Neurosci 2005 25 3002 3008 10.1523/JNEUROSCI.5295-04.2005 15772360 PMC6725129

[B25] Diana RA Yonelinas AP Ranganath C The effects of unitization on familiarity-based source memory: Testing a behavioral prediction derived from neuroimaging data J Exp Psychol Learn Mem Cogn 2008 34 730 740 18605864 10.1037/0278-7393.34.4.730 PMC2605011

[B26] Diana RA Van den Boom W Yonelinas AP Ranganath C ERP correlates of source memory: Unitized source information increases familiarity-based retrieval Brain Res 2011 1367 278 286 20965154 10.1016/j.brainres.2010.10.030 PMC3095515

[B27] Achim AM Bertrand MC Montoya A Malla AK Lepage M Medial temporal lobe activations during associative memory encoding for arbitrary and semantically related object pairs Brain Res 2007 1161 46 55 17604009 10.1016/j.brainres.2007.05.046

[B28] Preston AR Bornstein AM Hutchinson J Gaare ME Glover GH Wagner AD High-resolution fMRI of content-sensitive subsequent memory responses in human medial temporal lobe J Cogn Neurosci 2010 22 156 173 10.1162/jocn.2009.21195 19199423 PMC2854293

[B29] Awipi T Davachi L Content-specific source encoding in the human medial temporal lobe J Exp Psychol Learn Mem Cogn 2008 34 769 18605867 10.1037/0278-7393.34.4.769 PMC2938959

[B30] Liang JC Wagner AD Preston AR Content representation in the human medial temporal lobe Cereb Cortex 2013 23 80 96 10.1093/cercor/bhr379 22275474 PMC3513952

[B31] Watson HC Wilding EL Graham KS A role for perirhinal cortex in memory for novel object-context associations J Neurosci 2012 32 4473 4481 10.1523/JNEUROSCI.5751-11.2012 22457495 PMC6622046

[B32] Staresina BP Duncan KD Davachi L Perirhinal and parahippocampal cortices differentially contribute to later recollection of object- and scene-related event details J Neurosci 2011 31 8739 8747 10.1523/JNEUROSCI.4978-10.2011 21677158 PMC3128497

[B33] Staresina BP Davachi L Selective and shared contributions of the hippocampus and perirhinal cortex to episodic item and associative encoding J Cogn Neurosci 2008 20 1478 1489 10.1162/jocn.2008.20104 18303974 PMC2789239

[B34] Park H Shannon V Biggan J Spann C Neural activity supporting the formation of associative memory versus source memory Brain Res 2012 1471 81 91 22800807 10.1016/j.brainres.2012.07.012

[B35] Shafritz KM Gore JC Marois R The role of the parietal cortex in visual feature binding P Natl Acad Sci USA 2002 99 10917 10922 10.1073/pnas.152694799 PMC125073 12149449

[B36] Cusack R The intraparietal sulcus and perceptual organization J Cogn Neurosci 2005 17 641 651 10.1162/0898929053467541 15829084

[B37] Jackson O Schacter DL Encoding activity in anterior medial temporal lobe supports subsequent associative recognition Neuroimage 2004 21 456 462 10.1016/j.neuroimage.2003.09.050 14741683

[B38] Park H Rugg MD Neural correlates of encoding within- and across-domain inter-item associations J Cogn Neurosci 2011 23 2533 2543 10.1162/jocn.2011.21611 21254802 PMC3309035

[B39] Corbetta M Shulman GL Control of goal-directed and stimulus-driven attention in the brain Nat Rev Neurosci 2002 3 201 215 11994752 10.1038/nrn755

[B40] Corbetta M Patel G Shulman GL The reorienting system of the human brain: From environment to theory of mind Neuron 2008 3 306 324 18466742 10.1016/j.neuron.2008.04.017 PMC2441869

[B41] Lewis JW Beauchamp MS DeYoe EA A comparison of visual and auditory motion processing in human cerebral cortex Cereb Cortex 2000 10 873 888 10.1093/cercor/10.9.873 10982748

[B42] Renier LA Anurova I De Volder AG Carlson S VanMeter J Rauschecker JP Multisensory integration of sounds and vibrotactile stimuli in processing streams for “what” and “where” J Neurosci 2009 29 10950 10960 10.1523/JNEUROSCI.0910-09.2009 19726653 PMC3343457

[B43] Gottlieb LJ Uncapher MR Rugg MD Dissociation of the neural correlates of visual and auditory contextual encoding Neuropsychologia 2010 48 137 144 10.1016/j.neuropsychologia.2009.08.019 19720071 PMC2795095

[B44] Mayes A Montaldi D Migo E Associative memory and the medial temporal lobes Trends Cogn Sci 2007 11 126 135 10.1016/j.tics.2006.12.003 17270487

[B45] Quamme JR Yonelinas AP Norman KA Effect of unitization on associative recognition in amnesia Hippocampus 2007 17 192 200 10.1002/hipo.20257 17203466

[B46] Bar M Aminoff E Cortical analysis of visual context Neuron 2003 38 347 358 10.1016/S0896-6273(03)00167-3 12718867

[B47] Ross RS Slotnick SD The hippocampus is preferentially associated with memory for spatial context J Cogn Neurosci 2008 20 432 446 10.1162/jocn.2008.20035 18004952

[B48] Aminoff E Gronau N Bar M The parahippocampal cortex mediates spatial and nonspatial associations Cereb Cortex 2007 17 1493 1503 16990438 10.1093/cercor/bhl078

[B49] Ashburner J Friston K Nonlinear spatial normalization using basis functions Hum Brain Mapp 1999 7 254 266 10.1002/(SICI)1097-0193(1999)7:4<254::AID-HBM4>3.0.CO;2-G 10408769 PMC6873340

[B50] Friston KJ Penny W Phillips C Kiebel S Hinton G Ashburner J Classical and Bayesian inference in neuroimaging: Applications Neuroimage 2002 16 484 512 10.1006/nimg.2002.1091 12030833

